# Compressive Behavior of Fully Grouted Concrete Bond Beam Block Masonry Prisms

**DOI:** 10.3390/ma18112589

**Published:** 2025-06-01

**Authors:** Fei Zhu, Yongcheng Hang, Fenglai Wang, Shengbao Wang

**Affiliations:** 1 School of Civil Engineering, Harbin University of Science and Technology, Harbin 150080, China; 2 School of Civil Engineering, Harbin Institute of Technology, Harbin 150090, China

**Keywords:** compressive behavior, bond beam concrete blocks, masonry prisms, stress-strain relationship, monotonic and cyclic loading

## Abstract

This paper presents a study on the uniaxial compressive behavior of fully grouted concrete bond beam block masonry prisms. A total of 45 (i.e., 9 hollow and 36 fully grouted) specimens were tested, and the failure modes and initial crack were reported. The effects of block strength, grout strength, and loading scheme on the compressive strength of the fully grouted prism were discussed. The results show that the compressive strength of bond beam block prisms increased with an increase in grouting, while they were less affected by the block strength; the peak strength of the grouted block masonry was, on average, 35.1% higher than the hollow masonry prism. In addition, although the specimens’ strength was lower under cyclic compression than under monotonic compression loading, the difference in their specified compressive strength was statistically insignificant. The stress–strain curve of block masonry under uniaxial compression was also obtained. Through nonlinear fitting, the compressive stress–strain relationship of grouted masonry, considering masonry strength parameters, was established, which demonstrated alignment with prior experimental studies. This study not only provides a strength calculation method for grouted masonry structures using high-strength blocks in the code for the design of masonry structures in China but also offers a dedicated stress–strain curve for precise finite element analysis and the design of masonry structures.

## 1. Introduction

The compressive strength of masonry is the most important material property for the design of structural masonry. For fully grouted concrete block masonry, behavior and strength under compressive loading is a fundamental research topic, and it is influenced by mortar strength [1,2,3,4,5], block strength [6,7], grouted concrete strength [8,9,10], height-to-thickness ratio [7,11], loading scheme, and bond pattern [12]. Drysdale and Hamid carried out a series of experimental tests and found that mortar strength did not have much effect on the compressive capacity of fully grouted concrete block masonry, in accordance with other researchers [1,5,13]. Fahmy and Ghoneim used finite element analysis and stated that the increase in block unit strength increased the strength of grouted prisms, but this was not obvious when the block strength reached a certain level [14], while increasing the grout strength only resulted in a small increase in prism strength [1,15]. Boult pointed out that an increase in the height-to-thickness ratio of the prism does decrease its compressive strength. In addition, a prism height-to-thickness ratio between 5 and 10 is assumed to have no effects on the difference in strength; thus, a height-to-thickness ratio of 5 was used as a base point in CSA S304.1-04 [16]. Dhanasekar et al., based on experimental tests, investigated fully grouted prisms with full bedding mortar, finding that there was a consistent stress–strain envelope between monotonic and cyclic compression loading, while the face-shell bedded masonry showed the effect of load cycle with an approximately 20% reduction in peak stress compared to monotonic stress–strain curves [9,17]. Kingsley and Atkinson concluded that a running bond pattern results in an 11% decrease as opposed to a stack bond in compressive strength for grouted prisms [18]. However, Hamid determined that, for grouted prisms, there was no significant effect of the bond type on compressive strength. Admittedly, in spite of stack prisms being easy to handle in the laboratory, the running bond pattern including vertical joints is closer to the actual compression state of masonry prisms [15]. It should be noted that the conclusions mentioned above were based on masonry with American stretchers and half-unit blocks.

Bond beam blocks are an adaptation of the American stretcher unit to lay out transverse rebars that, when combined together, form infill concrete. Reinforced grouted concrete bond beam block masonry is widely used as anti-seismic shear walls due to its efficient construction and economic benefits; this has been promoted as a green building structure system in China [19,20,21]. In addition, new systems of reinforced grouted concrete block masonry have been innovated by changing the block pattern and reinforced type to enhance their compression, shear, ductility, and anti-seismic behavior [22,23,24,25,26,27,28]. Currently, horizontal reinforcement in masonry construction includes joint reinforcement and reinforcing bars. Joint reinforcement is easy to implement in construction and is well understood, while the diameter of the rebar is restricted by the thickness of the mortar layer usually in ladder or truss types when subjected to severe lateral force [29,30]. To date, neither has a compressive strength formula for high-strength block masonry (>20 MPa) been specified in the code for the design of masonry structures nor has the corresponding stress–strain curve been provided. Thus, adopting rational and precise mechanical performance parameters becomes imperative to ensure structural safety in computational analyses of such systems. Therefore, in an experiment tested in a universal testing machine, the effects of block strength, grout strength, and loading pattern on the compressive behavior of this block masonry were systematically investigated.

## 2. Materials and Experimental Programs

### 2.1. Materials Properties

**Figure 1:**
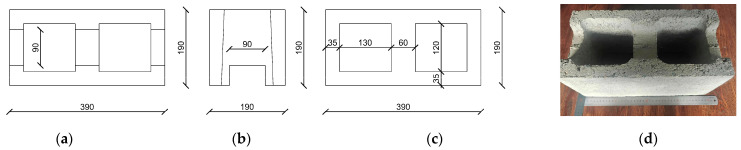
Bond beam block: (**a**) top surface dimension line (unit of mm); (**b**) bottom surface dimension line(unit of mm); (**c**) side surface dimension line (unit of mm); (**d**) sample photo.

The bond beam block has the same overall dimensions as the stretcher unit, designed to receive horizontal reinforcement and grout, with the detailed dimensions listed in Figure 1. Hollow concrete stretcher blocks were produced by the BESSER company in V3-12K series; the bond beam blocks are knockout 90 × 60 mm (height × width) stretcher units of the web, and all types of blocks were supplied by a block production factory in southeast China. Three types of block-fill grout were ordered from a ready-mix supplier. The properties of the different blocks and concrete adopted for construction of the masonry specimens are presented in Table 1 and Table 2, respectively. A moderate composition of mortar with a compressive strength of 15.5 MPa was cast for all prisms.

Köksal et al. recommended that grout strength equal to or greater than that of the block could prevent a sharp decrease in the prism’s ultimate load capacity of the prism [13]. Furthermore, considering deformation compatibility, the grout concrete strength should be at least 50% higher than the block strength in grouted masonry construction. Accordingly, this study utilized concrete with a higher strength than the blocks [31].

The block tests (Table 1) indicated a maximum coefficient of variation for the net-to- gross area ratio of only 0.016. The elastic modulus of the blocks increased from 16,533 to 20,746 MPa as their compressive strength rose from 25.73 to 31.18 MPa. The standard deviation of block compressive strength increased with higher compressive strength, whereas the standard deviation of the elastic modulus decreased as the modulus of elasticity increased.

As shown in Table 2, the concrete mix proportions are by mass, with the cement content normalized to 1.0. Additionally, all concrete types incorporated a copolymer superplasticizer at a mass proportion of 0.05%. Contrary to the block compressive strength results, the standard deviation of concrete compressive strength decreased as the compressive strength increased. Although the elastic modulus of concrete was determined from the deformation of the specimen height, the secant modulus between 50% and 70% of the ultimate stress increased with increasing compressive strength.

The design and nomenclature of the test specimens are listed in Table 3. Hollow concrete masonry prisms, constructed with the same moderate-strength mortar, were fabricated using three different stretcher block types: B1, B2, and B3. Five types of fully grouted masonry prisms were prepared: C2 grout was used with all three hollow prism types (B1, B2, and B3); C1 grout was used with B1 blocks; and C3 grout was used with B3 blocks.

### 2.2. Specimen Details

Testing was conducted on three types of hollow block masonry specimens built with stretcher blocks (HB1, HB2, and HB3) and five types of fully grouted block masonry specimens with bond beam blocks (G1B1, G2B1, G2B2, G2B3, and G3B3 series).

Bond beam block prisms are analogous to face-shell bedded prisms constructed with stretcher blocks, owing to the void in the mortar layer between the central block webs; related research conclusions can be cited in this context. In North America and Australia, face-shell mortar bedding is common practice due to its benefits of faster construction and reduced mortar consumption. Furthermore, Jia found that the strength improvement in prisms built with full bedding, compared to those with face-shell bedding, is statistically insignificant [32,33]. However, Ganesan and Ramamurthy discouraged the use of face-shell bedding in hollow prisms based on finite element analysis, which revealed high lateral tensile stress concentrations in the webs [32]. Furthermore, neither face-shell bedding nor bond beam blocks are specifically addressed in Chinese masonry design codes, test standards, or construction handbooks. Therefore, as bond beam hollow prisms are uncommon in practice and exhibit drawback mechanisms under compression, hollow prisms built with stretcher blocks were constructed for simplification in this study. However, their compressive strength values should be considered indicative when compared to grouted prisms, given the different failure conditions induced by grouting.

**Figure 2:**
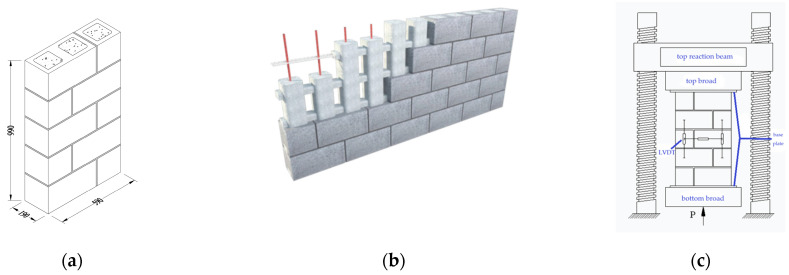
Specimen details: (**a**) dimensions of specimen (unit of mm); (**b**) schematic diagram bond beam walls; (**c**) test set up.

The typical dimensions of the test specimens are shown in Figure 2a; a height-to-thickness ratio of 5.0 was adopted to minimize its influence. Prisms constructed in a running bond pattern better represent wall behavior than stack-bonded prisms due to the presence of perpendicular joints in each course. A schematic diagram illustrating the constitution of fully grouted bond beam masonry walls, including potential horizontal and vertical reinforcement, is presented in Figure 2b.

Three specimens were cast for each hollow block masonry prism combination. A total of 36 fully grouted prisms, encompassing five types, were constructed. An average mortar joint thickness of 10 mm was maintained for all specimens. Grout was consolidated by a single pass of a 25 mm immersion vibrator in each cell and was not revibrated after initial settlement. All specimens were level-capped with gypsum plaster and cured for 28 days in the laboratory.

### 2.3. Testing Methodology

Uniaxial compressive strength tests on hollow and fully grouted prisms were conducted using a 10,000 kN capacity electro-hydraulic testing machine, capable of applying displacement with a measurement precision of 0.001 mm. Load was monitored by the machine with a measurement precision of 0.1 kN. The specimens were capped with two 25 mm thick steel plates at the top and bottom to ensure uniform distribution of axial stress during compression. To ensure full contact between the specimen and the bearing, a spherical bearing was bolted to the bottom platen of the machine. Test specimens were subjected to a constant displacement rate of 0.3 mm/min; displacement and corresponding load were recorded using an electronic data acquisition system [34]. The test set up is shown in Figure 2c.

**Figure 3:**
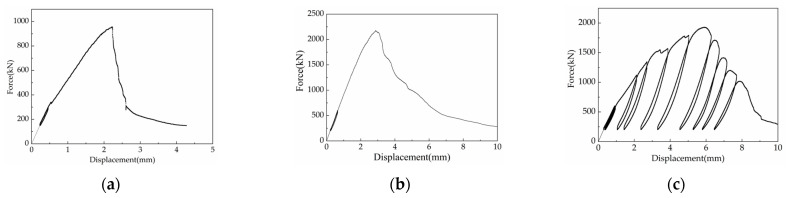
Loading–displacement curves: (**a**) hollow prism under monotonic loading; (**b**) grouted bond beam under monotonic loading; (**c**) grouted bond beam under cyclic loading.

In the first phase of testing, all three types of hollow prisms and 20 of the fully grouted prisms (across five types) were tested to failure at a uniform displacement rate, with each test lasting approximately 15 to 40 min. In the second phase, the remaining 16 fully grouted specimens (representing three types) were subjected to cyclic loading, with tests lasting from 50 to 120 min. Loading and unloading cycles were performed multiple times to analyze how the strength of the grouted prisms changed with different loading patterns. The general loading and unloading schemes, represented by load–displacement curves for monotonic and cyclic tests (with machine adjustments removed), are shown in Figure 3.

## 3. Results and Discussion

### 3.1. Failure Modes

**Figure 4:**
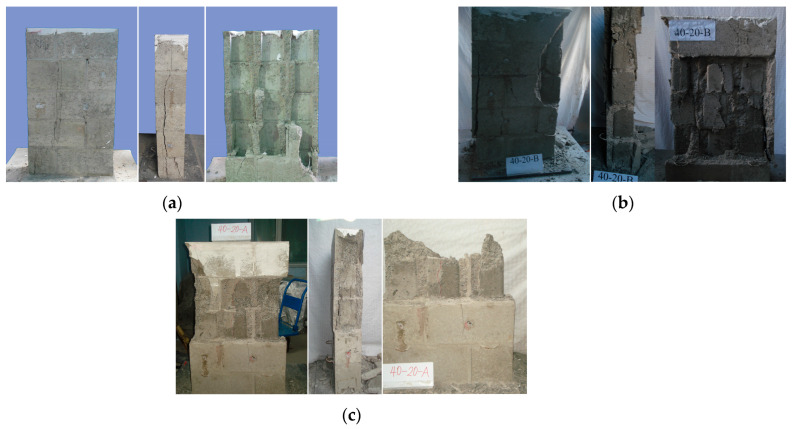
Failure modes: (**a**) hollow prism; (**b**) grouted bond beam to about 20% residual strength; (**c**) grouted bond beam to failure.

The failure modes observed for hollow prisms under monotonic compression and for grouted prisms under monotonic and cyclic compression are depicted in Figure 4.

Hollow concrete block masonry prisms under uniaxial compression typically failed by splitting with vertical cracks, as shown in Figure 4a. Visible cracking initiated in the block face shells on both sides at approximately 71–84% of the peak load (Table 3). Subsequently, the mortar joints crushed and cracks in the blocks propagated. Near the peak load, vertical cracking initiated in the middle of the transverse webs, attributable to the lower stiffness of the face-shell regions. Cracks propagated rapidly near the peak load, accompanied by mortar crushing. The main cracks continued to progress, with local failure occurring during the descending branch, indicative of a pure brittle failure. Subsequently, the prism separated into two independent panels. This failure mode was also observed by Barbosa [34].

For fully grouted bond beam block prisms under monotonic compression, initial visible vertical cracking was observed on the face-shells at approximately 76–83% of the average ultimate load (Table 3). As the load increased, these cracks widened and elongated, and additional cracks appeared on both the face-shells and webs of the blocks, primarily in the middle three courses. Face-shell spalling, caused by the expansion of the infill grout and mortar crushing, occurred near the ultimate strength, at which point cracks parallel to the loading direction were observed in the infill grout. During the descending branch, these grout cracks continued to widen but no new cracks appeared in the blocks. The specimens exhibited ductile failure, retaining approximately 20% of their ultimate strength as residual strength, and separated into individual columns under both monotonic and cyclic loading (Figure 4b). If displacement application continued, the infill grout could exhibit failure behavior similar to that of plain concrete (Figure 4c). Block splitting failure was also observed by Liang in research on H-block prisms [35]. Additionally, the specimens did not exhibit any strength recovery upon reloading, a finding also reported by Dhanasekar (see Figure 3c) [9]. This suggests that, despite the different time durations of monotonic and cyclic tests, the distinction in failure modes was insignificant, possibly due to the equivalent effective work done on the specimens.

### 3.2. Effect of Block Strength

Table 4 presents the measured compressive strengths. Three different types of hollow concrete blocks were used in this study, with block compressive strength to mortar compressive strength ratios varying from 1.66 to 2.01. For hollow concrete block masonry prisms under monotonic compression, the gross area strength increased from 7.23 to 9.94 MPa as the block compressive strength increased from 25.73 to 31.18 MPa. The standard deviation of the hollow prism compressive strength decreased as the constituent block compressive strength increased.

For fully grouted concrete block masonry prisms under monotonic compression, the influence of variations in both block strength and grouted concrete strength on masonry strength was investigated. When using the same grout strength (specimen series G2B1, G2B2, and G2B3), tests showed that, as block compressive strength increased from 25.73 to 31.18 MPa, the masonry strength initially increased from 20.17 to 21.14 MPa but then decreased to 19.50 MPa.

**Figure 5:**
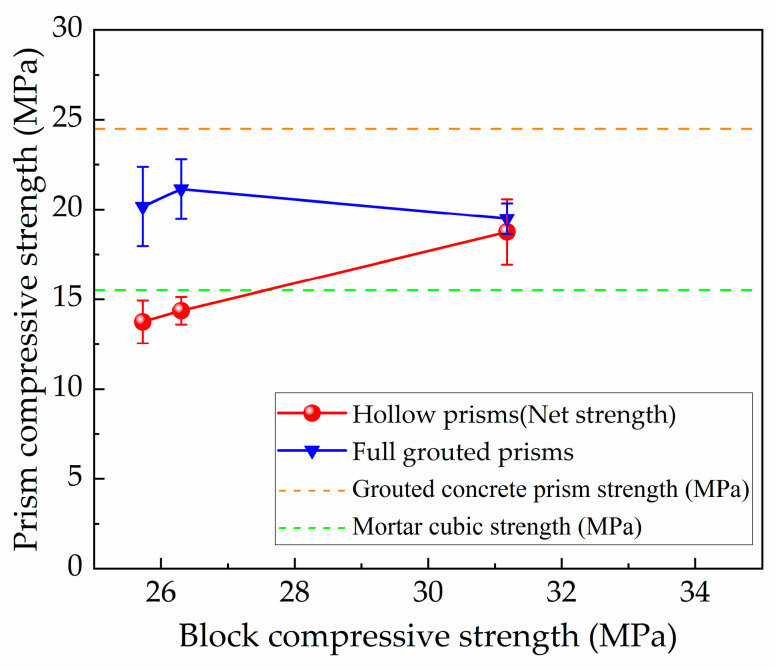
Prism strength versus block strength.

Figure 5 relates the compressive strength, based on net area, of hollow and fully grouted prisms compressed under monotonic loading to the compressive strength of block. The figure shows that increasing the block strength resulted in an increase in the prism strength for hollow prisms and a slight decrease in the prism strength for fully grouted prisms. For fully grouted prisms, the increase in block strength of 2.2% for type B2 compared to type B1 resulted in a corresponding increase in prism strength of 4.8%; then, an increase in the block strength of 21.2% from B1 to B3 resulted in a prism compressive strength decrease of 3.3%. Clearly, the effect of changing the strength of the block was less significant in fully grouted prisms than hollow prisms in this test. This is attributed to the effect of grout changing the failure mechanism from the hollow prisms governed by block splitting to the grouted prism governed by concrete crush, which is observed during the tests. Based on the experimental results, we can obtain that the peak strength of the grouted block masonry is, on average, 35.1% higher than the hollow masonry prism.

Therefore, since the block strength was not an extremely sensitive parameter, the remainder of the tests, namely specimen pattern G1B1, G2B2, and G3B3, seemed to be approximately the same block strength, and then they were simplified as the variation in grouted concrete strength.

### 3.3. Effect of Grout Strength

**Figure 6:**
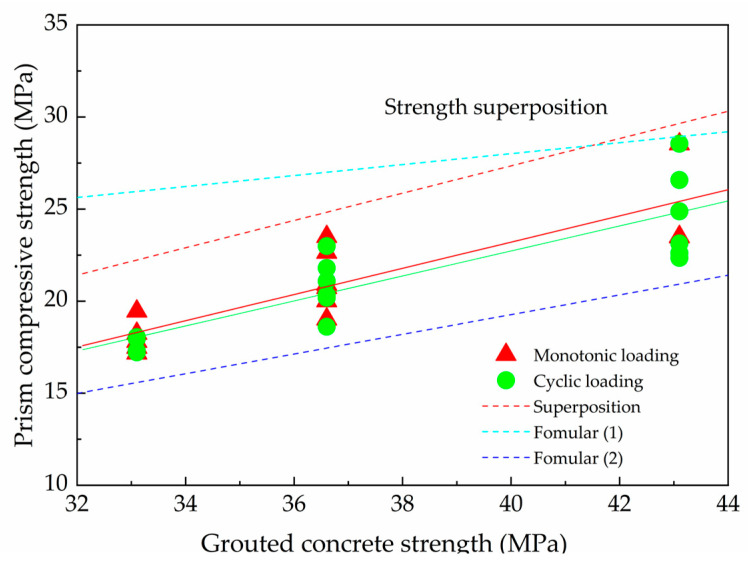
Prism strength versus grout concrete strength.

As shown in Figure 6, the red and green lines represent the least squares fit of the test data for grouted prisms under monotonic and cyclic loading, respectively. A 10.6% increase in concrete strength (type G2 compared to G1) resulted in corresponding prism strength increases of 17.3% (monotonic) and 18.1% (cyclic). Subsequently, a 30.2% increase in concrete strength (from G1 to G3) led to prism compressive strength increases of 39.6% (monotonic) and 40.0% (cyclic), respectively. These data indicate that, for running bond prisms with concrete strengths ranging from 33.1 to 43.1 MPa, grout strength has an approximately linear influence on the compressive strength of the grouted prisms under both monotonic and cyclic loading.

This is because increasing grout strength has two opposing influences on prism strength. First, increasing grout strength enhances the overall compression capacity of the prism. Second, it can reduce the vertical stress in the mortar, thereby decreasing the lateral confining stresses induced in the blocks by the mortar. Conversely, higher vertical stress in the grout leads to greater induced lateral tensile stresses in the block units due to grout confinement. In this study, the crack-to-peak stress ratio (Table 4) was observed to be approximately equal for grouted prisms and their corresponding hollow counterparts. It could be concluded that this ratio would be higher than that for hollow prisms made with bond beam blocks due to the influence of deep beam action leading to premature block failure. This phenomenon implies that the beneficial effect of increased grout strength (enhancing prism capacity) outweighs the detrimental effect of increased lateral tension in the blocks (attributable to initial cracking), leading to the observed linear relationship between prism strength and grout strength. This relation was also observed by Fahmy and Ghoneim when the mortar strength was less than the block and grout strength [14].

In Figure 6, the red dashed line represents the compressive strength calculated by simple superposition of the hollow prism strength and the grout concrete strength. It is evident that the actual compressive strength of grouted masonry is lower than that predicted by the superposition approach. The ratio of the superposition-predicted prism strength to the grout concrete strength is 0.74, while the ratio of the experimentally fitted prism strength to the grout concrete strength is 0.71 under monotonic loading. The difference between the superposition-predicted and experimentally fitted strengths appears relatively constant across the tested grout concrete strength variations.

The formula for calculating the compressive strength of grouted masonry in the Chinese code for the design of masonry structures is:

(1) $$f_{mg}=f_{m}+0.63\alpha f_{cu,m}\;f_{m}=0.46{f_{1}}^{0.9}(1+0.07f_{2})(1.1-0.01f_{2}),$$

Note: the factor $f_{mg}$ is compressive strength of grouted concrete block masonry, $f_{mg}$ is the average compressive strength of masonry, α is the ratio of the area of grouted concrete in concrete block masonry to the area of masonry, $f_{cu,m}$ is the average strength of grouted concrete, $f_{1}$ is the average compressive strength of block, and $f_{2}$ is the average compressive strength of mortar. Notably, the Chinese code does not provide a calculation formula for the compressive strength of grouted masonry when the block’s compressive strength exceeds 20 MPa.

For comparison with conventional block prisms, a formula established by Drysdale and Hamid based on experimental tests is also plotted in Figure 6 and presented as Formula (2).

(2) $$f_{mg}=\eta \cdot (1-K(1-\eta )\frac{\sigma_{cg}}{f_{mu}})\cdot f_{mu}+(1-\eta )\cdot \sigma_{cg},$$

Note: the factor $f_{mg}$ is compressive strength of grouted concrete block masonry, η is net to gross area ratio of the block, K is a coefficient reflecting the interaction between the block shell and the grouted core, $\sigma_{cg}$ is the grout compressive strength calculated from block molded prisms, which is 50% higher than steel molded prisms due to the absorption of water from the grout by the concrete masonry units, and $f_{mu}$ is the compressive strength of hollow concrete block masonry based on net area [35].

As shown in Figure 6, the mean compressive strength calculated using Formula (1) is higher than the experimental results; specifically, the calculated values are, on average, 30% higher than the measured values. This indicates that Formula (1) provides unsafe predictions and is unsuitable for calculating the masonry strength of high-strength grouted concrete blocks.

As depicted in Figure 6, the experimental results for bond beam blocks were higher than those predicted by Formula (2), implying that grout has a more significant influence on bond beam prisms than on conventional prisms, likely due to the higher proportion of grout in the bond beam specimens. For the compressive strength of grouted masonry with high-strength blocks, Formula (2) is more conservative and could serve as a supplementary provision to the Chinese code.

### 3.4. Loading Pattern

To analyze the influence of loading type on the prisms, the mean strength, specified strength, and the ratio of cracking load to peak load were calculated for both loading types. Table 5 summarizes the test results for masonry prisms under monotonic and cyclic loading, including individual and mean compressive strengths, specified compressive strength, and loading type ratios. The peak compressive strength for prisms under cyclic loading was lower than that under monotonic loading; for the G1B1, G2B2, and G3B3 series, the Kp ratio (cyclic mean to monotonic mean) was 0.98, 0.99, and 0.98, respectively. However, the coefficient of variation for compressive strength was higher under monotonic loading compared to the corresponding cyclic loading.

The specified strength is calculated by applying a statistical reduction (mean strength minus 1.645 times the standard deviation) to the mean strength of the test specimens. This statistical reduction factor of 1.645, assuming a normal distribution, aims to define a characteristic compressive strength that has a 95% probability of being exceeded by the actual strength of the masonry prism. Although the peak strength of specimens under cyclic loading was slightly lower than that under monotonic loading, from a structural design perspective using specified compressive strength, the Ks ratio in Table 5 indicates that the difference between prisms tested under monotonic and cyclic loading is statistically insignificant.

### 3.5. Comparison with the Fully Grouted Concrete Masonry Prisms Built with Stretcher Block

As suggested by the comparison with Formula (2) in Figure 6 (which is for conventional prisms), bond beam block prisms exhibited higher compressive strength normal to the bed joint. This may be attributed to an approximately 10% greater proportion of grouted concrete in bond beam prisms, which significantly influences their compressive behavior. Additionally, for strength parallel to the bed joint, Ring et al. experimentally investigated the effect of varying amounts of grout interruption on the compressive strength of fully grouted concrete block masonry [36]. Their study showed that compressive strength decreases as the amount of web interruption increases. Specifically, the bond beam blocks used in this paper with a web interruption of 87.5% exhibited higher strength compared to conventional block prisms with 100% web interruption. It should also be noted that this conclusion is applicable when there is a good strength match between the grouted concrete and the block.

However, in addition to construction benefits similar to those of face-shell bedded prisms, bond beam blocks with knockout webs are lighter in weight compared to standard stretcher units. Furthermore, in bond beam block prisms, the grout forms transverse concrete stripes connecting the vertical and horizontal cores, creating a more reliable connection than the block–mortar interfaces found in conventional prisms, which often represent a weak region in masonry. Thus, appropriate reinforcement within these concrete stripes could significantly improve both strength and ductility [15,37]. Accordingly, the use of bond beam blocks represents an improved fully grouted concrete block masonry system compared to conventional prisms and could be advantageous in high seismic regions.

### 3.6. Stress-Strain Curves

Recognizing the importance of accurately measuring stress–strain curves for block masonry, Priestley and Elder introduced a technique to derive these curves by adjusting the measured deformation relative to the overall specimen height [38]. This technique was developed during their investigation into the uniaxial compressive behavior of hollow and grouted block masonry. Specifically, corrections were applied to deformation data obtained from the testing machine’s platen-to-platen measurements. The procedure involved (1) subtracting the initial compaction deformation that occurred during preloading and accounting for any gap between the specimen and the press; (2) adjusting the ascending and descending segments of the load–displacement curve by, respectively, subtracting and adding the elastic stiffness of the press; and (3) incorporating an elastic unloading correction for the top and bottom block masonry affecting the central deformed region in the descending segment of the curve. The resulting stress–strain curves closely matched those obtained using direct displacement meter (e.g., LVDT) attachment methods, leading to the widespread adoption of their correction technique.

In the present experiment, the deformation processing technique proposed by Priestley and Elder was utilized to determine the stress–strain curves of the specimens [38]. Additionally, direct displacement meter attachment was employed to monitor and validate the deformation of the masonry components. During the preloading phase, the functionality of each attached displacement meter was verified, ensuring proper adhesion to the component surface. This verification step ensured that deformation readings increased linearly prior to cracking and that discrepancies among the meters remained within acceptable limits. Following cracking, continuous monitoring of displacement meter values revealed significant increases near cracked regions, while slower or even negative strain development was observed in uncracked areas. As the specimens approached their peak strength, clear distinctions in failure characteristics emerged between the hollow and grouted masonry. At this stage, further reliable deformation measurements using the attached displacement meters became difficult and were often discontinued. For specimens subjected to cyclic loading, the reverse loading point (minimum compressive load) was set at 200 kN to minimize the influence of equipment slack and improve testing efficiency.

**Figure 7:**
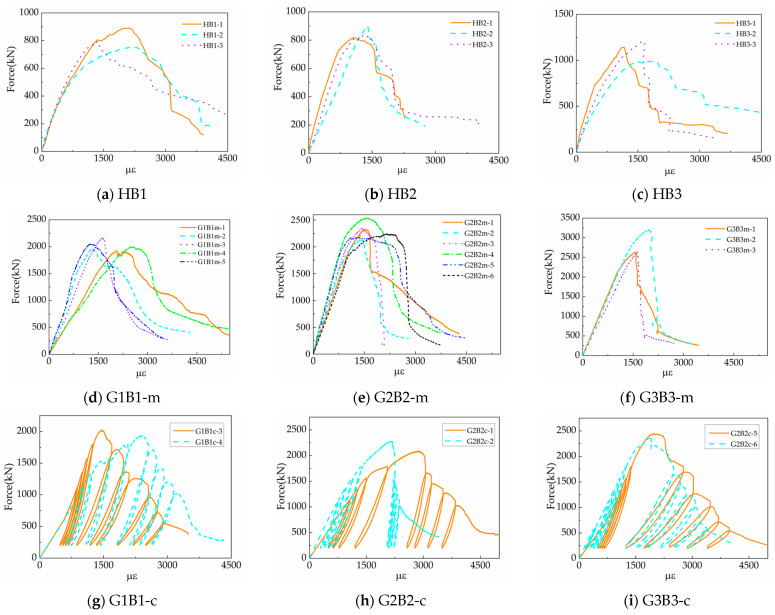
Experimental force–strain curves of masonry wallettes: (**a**) stress–strain curve of HB1 under monotonic loading; (**b**) stress–strain curve of HB2 under monotonic loading; (**c**) stress–strain curve of HB3 under monotonic loading; (**d**) stress–strain curve of G1B1 under monotonic loading; (**e**) stress–strain curve of G2B2 under monotonic loading; (**f**) stress–strain curve of G3B3 under monotonic loading; (**g**) the stress–strain curve of G1B1 under cyclic loading; (**h**) the stress–strain curve of G2B2 under cyclic loading; (**i**) the stress–strain curve of G3B3 under cyclic loading.

The load–strain curves for the various types of hollow concrete stretcher block masonry prisms and fully grouted concrete bond beam block masonry prisms tested are shown in Figure 7.

As shown in Figure 7, the tests yielded relatively ideal load–displacement curves, which were processed to obtain the load–strain curves of the specimens. The load–strain curves for both hollow and grouted masonry specimens exhibit both hardening and softening segments. The peak load capacity of the grouted masonry is significantly higher than that of the hollow masonry. The hysteresis curves for grouted masonry specimens under cyclic loading demonstrate satisfactory performance, with residual strain progressively increasing in proportion to the applied strain amplitude.

Given that a stress–strain curve equation for block masonry is not provided in the code for design of masonry structures and referencing the segmented stress–strain curve form for concrete in said code, a segmented stress–strain curve for block masonry was fitted using the least squares method. The resulting equation is expressed as Formula (3), where k_0_ is an adjustment coefficient for the descending branch, related to the masonry strength grade, and k_1_ is a peak adjustment coefficient.

(3) $$\frac{\sigma }{f_{m}}=\left\{\begin{array}{c} -0.6\cdot (\varepsilon /\varepsilon_{m}{)}^{2}+1.6\cdot \left(\varepsilon /\varepsilon_{m}\right)\;\;\;\;\;\;\;\;\;\;\;\;\varepsilon \leq \varepsilon_{m}\\ k_{1}\cdot \frac{-0.18\cdot \left(\varepsilon /\varepsilon_{m}\right)+0.82}{(\varepsilon /\varepsilon_{m}{)}^{2}-k_{0}\cdot (\varepsilon /\varepsilon_{m})+1.64}\;\;\;\;\;\varepsilon >\varepsilon_{m} \end{array}\right.$$

$k_{0}=0.44\cdot \sqrt{f_{m}}$, $k_{1}=4.13-1.56\cdot k_{0}$, $f_{m}$ is the peak stress of the masonry, $\varepsilon_{m}$ is the peak strain of the masonry, σ is the masonry stress, and ε is the masonry strain.

**Figure 8:**
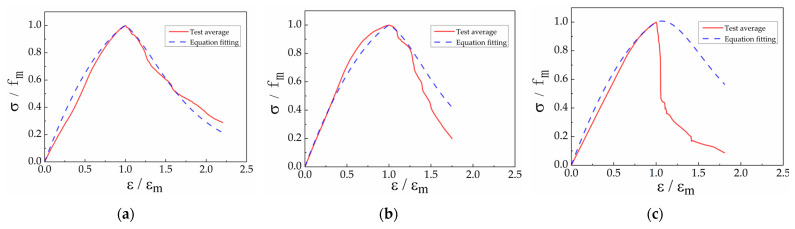
Comparison between test results and formula prediction: (**a**) G1B1 monotonic loading; (**b**) G2B2 monotonic loading; (**c**) G3B3 monotonic loading.

The average experimental curve and the equation-fitted curve for each test type are shown in Figure 8.

From the graph, the correlation coefficients (R^2^) between the fitted curves and the average experimental curves for G1B1, G2B2, and G3B3 are 0.981, 0.949, and 0.513, respectively. It is evident that the equation effectively describes the axial compression stress–strain curves of block masonry with different strength grades. For the G3B3-type block masonry, which exhibited high compressive strength, the descending branch of the stress–strain curve drops sharply. Further experimental research on this type of specimen is warranted.

### 3.7. Model Comparison

**Figure 9:**
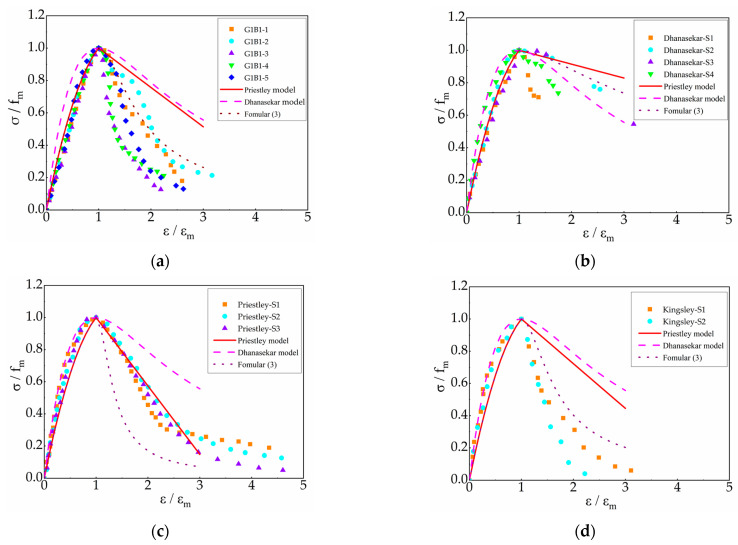
Comparison between test results and stress–strain models: (**a**) experiment and model prediction in this paper; (**b**) Dhanasekar test and model prediction; (**c**) Priestley test and model prediction; (**d**) Kingsley test and model prediction.

A total of five groups of previously published stress–strain curves were analyzed for comparison: three datasets from Priestley, two from Kingsley, and four from Dhanasekar. The model proposed in this study was compared with the Priestley and Dhanasekar models, with the results presented in Figure 9.

As illustrated in Figure 9, the proposed model, Dhanasekar’s model, and Priestley’s model all demonstrate close agreement with experimental data in simulating the ascending branch of the stress–strain curve, exhibiting insignificant discrepancies among them. For the descending branch, Priestley’s model aligns well with its own experimental data. However, when applied to other datasets, the slope of its predicted descending curve is flatter (less steep) than the experimentally measured values. Consequently, under identical ductility conditions, the residual strength predicted by Priestley’s model exceeds the experimental results. Similarly, Dhanasekar’s model closely matches its original experimental data but exhibits larger deviations than Priestley’s model when predicting other datasets. The proposed model (Formula (3)) demonstrates excellent consistency with the experimental data from the current study, as well as with the Dhanasekar and Kingsley test data. However, when predicting Priestley’s test data, the ductility represented by the proposed model’s curve is lower than the measured values. Comprehensive comparisons reveal that the proposed model provides a closer fit to experimental results, particularly in the descending branch, indicating its superior capability to characterize the stress–strain behavior of grouted masonry.

## 4. Conclusions

In this study, the compressive strength of fully grouted concrete bond beam block masonry prisms and corresponding hollow prisms was investigated through axial compression experiments. The test results revealed the following conclusions:

- Hollow concrete block masonry prisms under uniaxial compression failed by splitting with vertical cracks, whereas fully grouted bond beam block prisms failed by spalling of the block shells and crushing of the concrete core. The ratio of cracking load to peak strength varied from 0.71 to 0.85 for hollow prisms and from 0.76 to 0.83 for grouted prisms.
- The compressive strength of fully grouted concrete bond beam block prisms increased with increasing grout strength but was insignificantly affected by variations in block strength. Although a simple superposition of grout strength and hollow prism strength overestimates the actual prism strength, the tested strengths of bond beam prisms were higher than those typically reported for conventional grouted prisms (as inferred from comparison with existing models). For hollow concrete block prisms, increasing block strength led to an increase in prism strength. Grouted masonry exhibited, on average, 35.1% higher peak compressive strength compared to the net-area compressive strength of hollow masonry prisms under equivalent loading conditions.
- No strength recovery was observed when specimens were reloaded during cyclic testing. Although the average ratio of peak strength under cyclic loading to that under monotonic loading was 0.98, the specified compressive strength was found to be statistically insignificant between monotonic and cyclic compression.
- A two-stage polynomial stress–strain constitutive model for grouted masonry is proposed, demonstrating strong agreement with the experimental results. This model is applicable for simulating reinforced masonry shear wall structures and provides supplemental guidelines for Chinese masonry design codes.

Future work, combining experimental data and finite element simulation, should focus on establishing a meso-scale model (e.g., considering block–grout–mortar interfaces) to quantify deformation co-ordination and stress transfer mechanisms between different materials. This would address the insufficient simulation of interface failure under complex stress states in current models and improve the prediction accuracy of the mechanical performance of actual structures.

## Figures and Tables

**Table 1:** Properties of hollow concrete stretcher blocks.

Pattern	Net/Gross Ratio		Compressive Strength (MPa)		Secant Modulus at 40% of Ultimate Stress (MPa)	
	Average	Coefficient of Variation	Average	Standard Deviation	Average	Standard Deviation
B1	0.527 (10)	0.016	25.73 (15)	2.22	16533 (3)	1370
B2	0.528 (10)	0.010	26.30 (15)	2.42	18367 (3)	717
B3	0.530 (10)	0.005	31.18 (15)	4.62	20746 (3)	597

Note: numbers in parenthesis indicate number of specimens tested.

**Table 2:** Properties of concretes.

Pattern	Concrete Composition C^1^:S^2^:G^3^	w/c Ratio	28 Days Compressive Strength (MPa)		Secant Modulus Between 50% and 70% of Ultimate Stress (MPa)
			Average	Standard Deviation	
C1	1:2.0:3.5	0.6	33.1 (3)	1.05	4720
C2	1:1.9:3.1	0.6	36.6 (6)	0.87	6052
C3	1:1.4:2.6	0.5	43.1 (3)	0.46	8235

C^1^—cement; S^2^—sand; G^3^—stone. Note: numbers in parenthesis indicate number of specimens tested.

**Table 3:** Design and name of test specimen.

Block Type	Grout Type			
	Hollow	C1	C2	C3
B1	HB1 (3)	G1B1 (9)	G2B1 (3)	-
B2	HB2 (3)	-	G2B2 (12)	-
B3	HB3 (3)	-	G2B3 (3)	G3B3 (9)

Note: numbers in parenthesis indicate the specimens’ test number.

**Table 4:** Summary of test results for hollow and fully grouted masonry prisms.

Specimen Pattern	Block Strength (MPa)	Grouted Concrete Strength (MPa)	Monotonic Loading			Cyclic Loading	
			Specimens Tested	Crack to Peak Stress	Compressive Strength (MPa)	Specimens Tested	Compressive Strength (MPa)
HB1	25.73	-	3	0.84 (0.14)	7.23 (0.15)	-	-
HB2	26.30	-	3	0.82 (0.09)	7.64 (0.11)	-	-
HB3	31.18	-	3	0.71 (0.07)	9.94 (0.10)	-	-
G2B1	25.73	36.6	3	0.80 (0.21)	20.17 (0.11)	-	-
G2B3	31.18	36.6	3	0.76 (0.04)	19.50 (0.04)	-	-
G1B1	25.73	33.1	5	0.81 (0.10)	18.05 (0.05)	4	17.73 (0.03)
G2B2	26.30	36.6	6	0.83 (0.12)	21.14 (0.08)	6	20.84 (0.07)
G3B3	31.18	43.1	3	0.79 (0.08)	25.17 (0.13)	6	24.69 (0.10)

Note: numbers in parenthesis indicate the coefficient of variation.

**Table 5:** Compressive strength of prism under monotonic and cyclic loading.

Pattern and Type		Compression Strength (MPa)						Mean Strength (MPa)	Cov ^3^	Specified Strength (MPa)	Kp ^4^	Ks ^5^
G1B1	M ^1^	17.17	17.48	19.45	17.91	18.24	-	18.05	0.05	16.60	0.98	0.99
	C ^2^	17.31	17.96	18.42	17.22	-	-	17.73	0.03	16.79		
G2B2	M^1^	20.73	19.02	20.91	22.64	23.50	20.01	21.14	0.08	18.41	0.99	1.00
	C ^2^	18.61	20.35	20.18	22.99	21.80	21.09	20.84	0.07	18.37		
G3B3	M ^1^	23.48	28.53	23.50	-	-	-	25.17	0.12	20.38	0.98	0.99
	C ^2^	28.55	24.88	26.58	22.61	23.14	22.35	24.69	0.10	20.61		

Note: ^1^ Type M = monotonic loading, ^2^ Type C = cyclic loading, ^3^ Cov = the coefficient of variation of compressive strength, Ratio ^4^ Kp = the ratio of mean peak compression strength of monotonic to cyclic loading, ^5^ Ks = the ratio of specified compression strength of monotonic to cyclic loading.

## Data Availability

The original contributions presented in this study are included in the article; further inquiries can be directed to the corresponding author.
